# Profiling of Exome Mutations Associated with Progression of HBV-Related Hepatocellular Carcinoma

**DOI:** 10.1371/journal.pone.0115152

**Published:** 2014-12-18

**Authors:** Hyun Goo Woo, Soon Sun Kim, Hyunwoo Cho, So Mee Kwon, Hyo Jung Cho, Seun Joo Ahn, Eun Sung Park, Ju-Seog Lee, Sung Won Cho, Jae Youn Cheong

**Affiliations:** 1 Department of Physiology, Ajou University School of Medicine, Suwon, Republic of Korea; 2 Department of Gastroenterology, Ajou University School of Medicine, Suwon, Republic of Korea; 3 Graduate School of Biomedical Science, Ajou University School of Medicine, Suwon, Republic of Korea; 4 Institute for Medical Convergence, Yonsei University College of Medicine, Seoul, Korea; 5 Department of Systems Biology, The University of Texas M. D. Anderson Cancer Center, Houston, Texas, United States of America; The University of Hong Kong, China

## Abstract

Recent advances in sequencing technology have allowed us to profile genome-wide mutations of various cancer types, revealing huge heterogeneity of cancer genome variations. However, its heterogeneous landscape of somatic mutations according to liver cancer progression is not fully understood. Here, we profiled the mutations and gene expressions of early and advanced hepatocellular carcinoma (HCC) related with Hepatitis B-viral infection. Integrative analysis was performed with whole-exome sequencing and gene expression profiles of the 12 cases of early and advanced HCCs and paired non-tumoral adjacent liver tissues. A total of 293 tumor-specific somatic variants and 202 non-tumoral variants were identified. The tumor-specific variants were found to be enriched at chromosome 1q particularly in the advanced HCC, compared to the non-tumoral variants. Functional enrichment analysis revealed frequent mutations at the genes encoding cytoskeleton organization, cell adhesion, and cell cycle-related genes. In addition, to elucidate actionable somatic mutations, we performed an integrative analysis of gene mutations and gene expression profiles together. This revealed the 48 mutated genes which were differentially mutated with concomitant gene expression enrichment. Of these, *CTNNB1* was found to have a pivotal role in the differential progression of the HCC subgroup. In conclusion, our integrative analysis of whole-exome sequencing and transcriptome profiles could provide actionable mutations which might play pivotal roles in the heterogeneous progression of HCC.

## Introduction

Genomic heterogeneity of hepatocellular carcinoma (HCC) makes it difficult to characterize molecular pathogenesis and to develop efficient treatment modalities. During HCC development, aberrant genetic and epigenetic events occurred and accumulated, which provoked heterogeneous pattern of HCC progression. Previously, a number of sequence variations of HCC have been reported. For example, *TP53*, *CTNNB1*, and *AXIN1* have been reported to associate with the diverse progression pattern of HCC [1], [2]. Recently, the advance of high-throughput sequencing technology so called next generation sequencing (NGS) allowed us to profile mutations in a whole-genome scale. By applying NGS technology, genome-wide mutational spectra of HCC have been reported [3]–[7]. Numerous novel mutations such as *ARID1A* [8], *IRF2* [9], and *JAK1* [10] have been identified. In addition, the differential mutation spectrum of hepatitis B and C-related HCC has been studied [11]. By applying evolution models, stage-specific driver mutations (e.g., *CCNG1* and *P62*) have been noticed [12]. *LEPR* has been reported to be frequently mutated in the HCC-surrounded cirrhotic liver affecting tumor growth [13]. However, previous studies are still limited in the sample numbers, and the effect of mutations on the heterogeneous progression of HCC was not fully considered. Indeed, it is well known that the tumor grade is associated with the heterogeneous gene expressions and clinical outcomes of HCC [14]. Considering such studies, we sought to evaluate the role of mutation profiles with the tumor grader of HCC. In the present study, we performed whole–exome sequencing and compared the mutation profiles of HCC with different tumor grade of the early and the advanced cases. In addition, by performing integrative analysis of mutational profiles with their corresponding gene expression profiles, we sought the potential key regulators which are responsible for the heterogeneous progression of HCC. Our analysis could reveal prioritized candidates of functional and actionable mutations involved in the HCC progression, providing novel insight into the regulatory roles of mutation profile in the heterogeneous progression of HCC.

## Methods

### 1. Sample Preparation

A total of 12 cases of HBV-related HCC samples and paired adjacent non-tumoral tissues were obtained from the Ajou Human Bio-Resource Bank (AHBB), a member of the National Biobank of Korea, which is supported by the Ministry of Health and Welfare. The Institutional Review Board of Ajou University Hospital at Korea has approved this study, and waived the need for informed consent from donors.

### 2. Exome Capture and High-Throughput Sequencing

Whole exome sequencing was performed using the 12 cases of frozen HCC tissues. For targeted exome capture, Illumina TruSeq exome enrichment kit was used with the given protocol. The captured samples were sequenced as 110 bp paired-end reads using Illumina GAIIx with the average coverage of 30×. The 75 raw read bases from 5′ end start position were used trimming out the remaining 3′ sequences because the 10^th^ percentile of quality scores in each sample is less than 20. The trimmed reads were mapped to hg19 human reference genome using Burrows-Wheeler Alignment tool (BWA) [15] with default parameters. The mapping quality of the resulting.sam files was inspected, and those with zero quality were filtered out to reduce the false positive mapped reads. The PCR duplicates were identified and removed by using the Genome Analysis Toolkit (GATK) [16]. Then, local realignments of indels were performed using GATK local realignment walker [17], and the read quality was normalized using the GATK recalibration walker. Quality filter was performed using GATK unified genotyper with filter options of Hard to validate (MQ0> = 4 and MQ0/DP>0.1), Low coverage (DP <5), Low quality (QUAL <50.0), and Low quality-by-depth (QD <1.5). Each variant was annotated by using ANNOVAR [18]. Validation of the identified mutations was performed by Sanger (capillary) sequencing method.

### 3. Gene Expression Profiling

Total RNA was extracted by using the mirVana total RNA extraction kit (Ambion, Austin, TX, U.S.A) according to manufacturer's instruction and amplified by using Illumina TotalPrep 96 RNA Amplification Kit (IIlumina). Gene expression profiling was conducted with Illumina HumanHT-12 v4 Expression BeadChip kit. Microarray hybridization, image acquisition and processing were performed according to the manufacturer's guidelines. The raw data were log2 transformed, quantile normalized, and centered to each array mean for further analyses.

### 4. Functional Enrichment Analysis of Gene Sets

The enriched functions in the mutated gene sets were analyzed using Gene Ontology (GO) categories and KEGG database implemented in DAVID software [19]. Conservative statistical significance of the enrichments was estimated by EASE scores from modified Fisher's exact T-test P-values. For the gene expression profile analysis, the functional enrichment of gene sets in individual patient was determined by applying Kolmogorov-Smirnov (KS) test. For each individual gene expression profile, the directional *P*-values for the estimates D+ and D- were calculated by KS-test, and the enrichment score for a given signature was calculated as -log_10_ (*P*-value) as described previously [20]. The GO gene sets with more than ten genes were considered for analysis. All the statistical computation was performed using R software (http://www.r-project.org).

## Results

### 1. Identification of tumor-specific and non-tumoral mutations

Whole-exome captured sequencing was performed on the 6 cases of early HCC with tumor grade I or II, and the 6 cases of advanced HCC with grade III or IV, and the paired non-tumoral tissues. Clinical and pathological features of the specimens were described in S1 Table.

**Figure 1:**
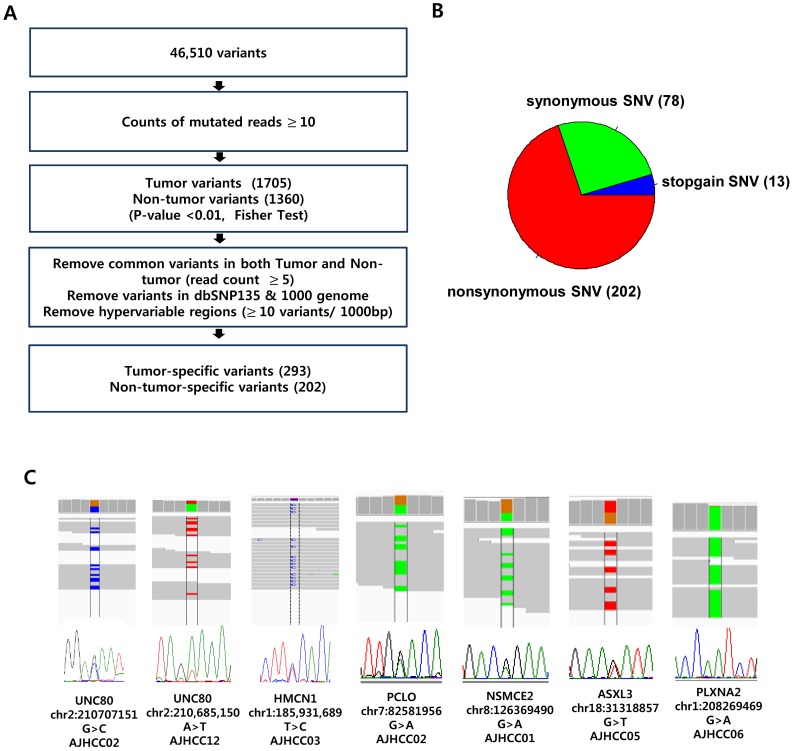
Identification of tumor-specific and non-tumoral variants. **A.** The flowchart for algorithms identifying tumor-specific and non-tumor-specific variants are shown. **B.** Distribution of somatic tumor-specific variants in HCC is shown. **C.** The tumor-specific variants are validated by Sanger sequencing method, and their read alignments are evaluated by Integrated Genome Browser (IGV) software.

Previously, it has been noticed that the current high-throughput NGS technologies still have limits because of substantial error rates of false base calls even with deep coverage profiling of sequence leads [21], [22]. Moreover, intra-tumoral heterogeneity of the mutations may lead to false negative calls especially in case of the use of small amount of tissues for sequencing experiment [23]. Thus, a stringent and careful variant calling pipeline might be required to reduce the false positivity of sequencing errors even for deep coverage data. With respect to this, we built an optimized pipeline for tumor-specific variant calls from our exome-seq data. First, following the classical sequence alignment pipeline using bwa and Genome Analysis Toolkit (for details see Methods), a total of 131,947 variants were mapped to human genome (hg19). Of these, we found a total of 46,510 exonic variants which were mapped with at least ten reads. Next, we tried to identify the most probable tumor-specific and non-tumoral tissue-specific variants, respectively. To estimate the tumor-specificity and/or non-tumor-specificity of the variants, we first calculated the odds between the number of variant counts of the paired tumor and non-tumor tissues (Fisher exact t-test, *P*<0.01), which yielded 1,705 tumor-specific and 1,360 non-tumor-specific variants, respectively. The non-tumoral variants were regarded as the background mutations for each patient. The variants called both in tumor and non-tumor tissues were considered as germline variants or non-specific calls, thus, we filtered them by applying more stringent filtering criteria of read counts ≥5 in the paired tissues. Then, to avoid false variant calls generated by possible local mis-alignment errors, the hyper-mutable regions with more than ten variants within a 1,000 base pair-sized window were also removed. Previously, kataegic foci with local enrichment of C>T mutations have been noticed in various cancers [24], [25]. However, the filtered hyper-mutable regions were not likely to be kataegis because the C>T enrichment was not observed. After removing the known SNPs (dbSNP135 and 1000 genome), we finally obtained 293 tumor-specific and 202 non-tumor-specific variants, which we used for further analyses. The details of the filtering pipeline were shown in **Fig. 1A** **.**

### 2. Genomic landscape of the tumor-specific variants

The 293 tumor-specific mutations include 202 nonsynonymous, 78 synonymous variants, and 13 codon stop-signaling variants (**Fig. 1B**). Previously, the synonymous variants so called silent mutations have been noticed to play driver roles in cancer development and progression [26], [27], therefore we did not exclude the synonymous variants in the subsequent analysis. Of the tumor-specific mutations, 148 genes including *CTNNB1*, *TTN*, *SETD2, ALK* have been previously observed in HCC from Catalogue Of Somatic Mutations In Cancer database (COSMIC, http://www.sanger.ac.uk/genetics/CGP/cosmic, v68) [28]. However, some of the well-known recurrent mutations such as *TP53*, *ARID1*, and *AXIN1* genes [11], [29] were not observed in our data, which might be due to small sample size and the application of high stringent criteria for variant calling. Of the tumor-specific mutations, the recurrent mutations with more than two observations were found in the 9 genes encoding *TCHHL1, PLXNA2, UNC80, CEP85L, PCLO, NSMCE2, ASXL3, SULF1*, and *MARS*. The frequencies of the tumoral and non-tumoral variants in each patient are highly variable ranging from 8 to 99, and 1 to 147, respectively. There was no significant difference of variant frequency between the groups of early and advanced HCCs (**S1 Figure**). We validated some of these variants by performing Sanger sequencing analysis and manual visualization of the sequence reads using Integrated Genome Viewer (IGV) [30] (**Fig. 1C**). This may support that our stringent variant calling can identify valid mutations despite of the relative low coverage of the sequencing reads. The list of tumor-specific and non-tumor-specific variants was summarized in **S2 Table**.

**Figure 2:**
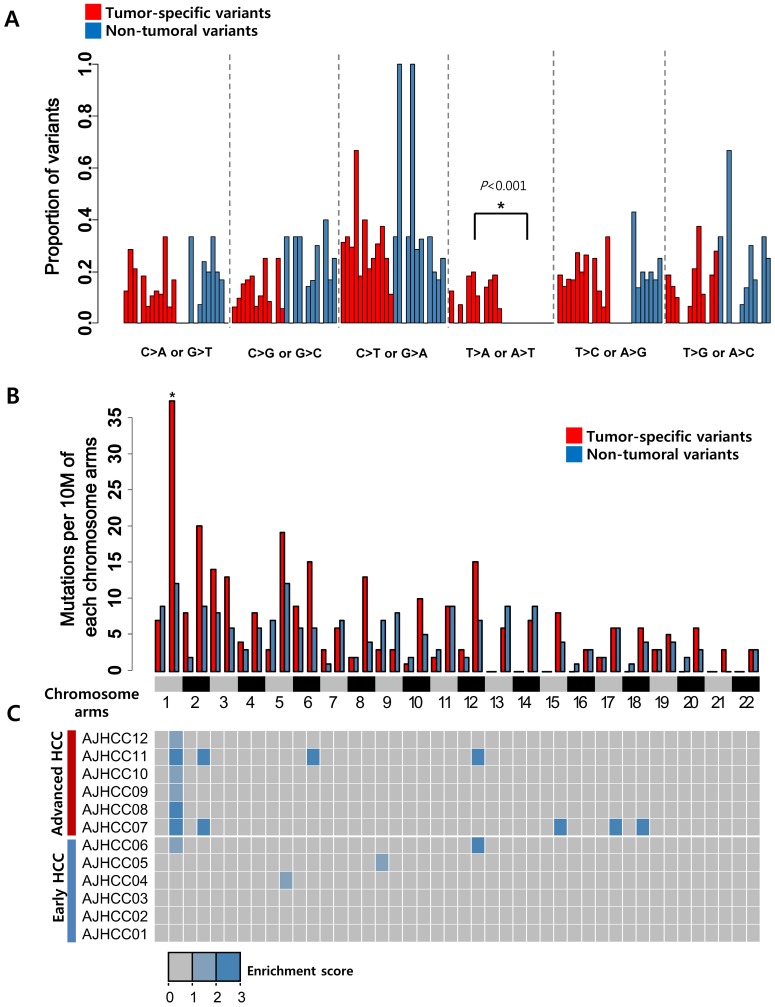
The mutation spectra of tumor-specific variants. **A.** Mutation spectrum of tumor-specific and non-tumor-specific variants are shown. **B.** The observed numbers of mutations per 10 million bases in each chromosome arm are plotted for tumor-specific and non-tumoral variants, respectively. Statistical analysis compared the occurrence of tumor-specific variants with that of non-tumoral variants. (*P<0.01, **P<0.001). **C.** Heatmap indicates the enrichment scores of tumor-specific variants which calculated as the odds ratios of the numbers of variants in each chromosome arm against those of outside the chromosome arm in each patient. The enrichment score less than 1 was truncated to zero.

Next, we examined the genomic patterns of the tumor-specific variants. Previously, the C:G>T:A transition is thought as a characteristic mutational signature of HCV-associated HCC [4]. However, another report has shown that the C:G>T:A transition was commonly found in HBV-associated HCC [5]. Consistently, we observed that the frequent C:G>T:A transition in both tumor-specific and non-tumoral variants. The prevalence of C:G>T:A transition was thought to associate with the aflatoxin B1 exposure[5]. However, the Korean patients in our study are not likely to relate with aflatoxin B1 exposure. Therefore, further elaboration might be required to validate and to explain the cause of the prevalence of the C:G>T:A transition. All the T:A>A:T transversions were found as tumor-specific variants (Wilcox test, p<0.001, **Fig. 2A**), which were consistent with the previous study regardless of different use of variant calling algorithms and patient cohorts[5].

We next examined the existence of any preferential site of the tumor-specific variants. Remarkably, we found that the tumor-specific somatic variants were significantly enriched at chromosome 1q compared to those in the non-tumoral tissues (Fisher's exact test, *P* = 0.009, **Fig. 2B**). The enriched mutations at 1q are not related with the size of the chromosome arms, because the enrichment scores of mutations were calculated by comparing to the number of mutations at the same chromosome arms of the non-tumoral tissues and the entire number of mutations in the whole genome of each patient. To confirm this finding in individual samples, we evaluated the mutation enrichment of chromosome arms for each sample data. Considering the individual variations of the background non-tumoral mutations, the enrichment of the tumor-specific variants was normalized with that of the non-tumor specific variants. In detail, the mutation enrichment score (ES) for each chromosome arm for each patient (j) was estimated by the odds ratios of the numbers of somatic variants (N) in each chromosome arm (i) against those of outside the chromosome arm (-i). 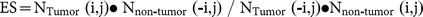


By calculating the enrichment scores of each chromosome arm, we found that the tumor-specific variants at 1q was significantly enriched in the advanced HCC group compared to early HCC group (**Fig. 2C**). This result suggests that the frequent mutations at 1q might be actionable with the association of the aggressive HCC progression. The genes with the enriched mutations at 1q encoded prevalently the protein and ion transport-related functions (e.g. *KIFAP3*, *MIA3*, *STX6*, *TOMM40L*, *CACNA1E*, and *RYR2*). We also evaluated the spectrum of the non-tumoral variants. The non-tumor-specific variants included 147 nonsynonymous variants, 49 synonymous ones, and 6 codon stop-signaling ones appearing similar distribution of the tumor-specific variants (**S2A Figure**). However, the chromosomal enrichment at 1q was not observed in non-tumoral mutations, suggesting that the chromosome 1q enrichment is tumor-specific observation (**S2B Figure**).

Previously, the somatic variants have been reported to occur in association with specific neighbor sequences. For example, A>C transversions at AA dinucleotide were reported to be commonly found in esophageal cancer [31]. With respect to this, we examined the sequence patterns of the flanking regions of the mutated positions by applying sequence logo algorithm which visualize the position-specific probability of the sequence variations [32]. However, we could not observe any base prevalence of the flanking sequences (**S3 Figure**).

### 3. Tumor-specific mutations reveal functional enrichment

Next, we evaluated the functional enrichment of the tumor-specific mutations because the enriched mutations are likely to be functional. The tumor-specific variants were found in the 280 genes, while the non-tumoral variants were found in the 198 genes, respectively (**S4A Figure**). Overall, the genes with tumor-specific mutations showed functional enrichment of cytoskeleton organization, cell adhesion, ion and protein transport, and transcription-related genes (**Table 1**). Noticeably, the genes coding cytoskeleton organizations showed the most prevalent enrichment (*P* = 0.0059), suggesting the enriched mutations of cytoskeleton genes play key roles in cancer development or progression. In addition, pathway analysis using KEGG database demonstrated a significant enrichment of cell cycle pathway (*P* = 0.005) (**S4B Figure**). Such associations of the enriched mutations with cancer aggressiveness implies a pivotal role of tumor-specific mutations in cancer development and progression. The non-tumoral mutations also showed the enrichment of cell cycle-related genes (**S3 Table**), although the genes were different from the genes with tumor-specific mutations. Only the 29 genes were overlapped between the genes with tumor-specific variants (10%) and the genes with non-tumor-specific variants (14.5%) (**S4C Figure**). This may suggest that the cell cycle-related genes might be highly mutable regardless of tumor and non-tumor tissues even though different genes being involved. In addition, the chromatin regulators have been noticed to be frequently mutated in HCC [9], [11]. Congruently, we also found the mutations of chromatin regulation-related genes including *XRCC5, RAD51C, HIST1H2BD, SETD2, TTN, HIST1H2AL, BAZ2A, MYSM1*, and *SUPT6H*, but no statistical significance was observed by the functional enrichment analysis.

**Table 1:** Functional categories of the tumor-specific mutations.

Category	Term	Gene counts	EASE score (P-Value)
cytoskeleton organization	cytoskeleton organization	15	5.9×10^−03^
	actomyosin structure organization	4	8.2×10^−03^
cell adhesion	cell adhesion	19	1.7×10^−02^
	calcium-dependent cell-cell adhesion	3	4.6×10^−02^
cell cycle	hsa04110: cell cycle (KEGG)	8	5.5×10^−03^
ion transport	calcium ion transport	8	5.4×10^−03^
	transmembrane transport	16	2.4×10^−02^
protein transport	vesicle-mediated transport	18	5.8×10^−03^
	protein localization	23	1.2×10^−02^
	intracellular transport	18	1.9×10^−02^
	protein transport	19	3.6×10^−02^
transcription initiation	transcription initiation from RNA polymerase II promoter	5	1.9×10^−02^

### 4. Differential tumor-specific variations of early and advance HCC are related with the transcriptional deregulation

**Figure 3:**
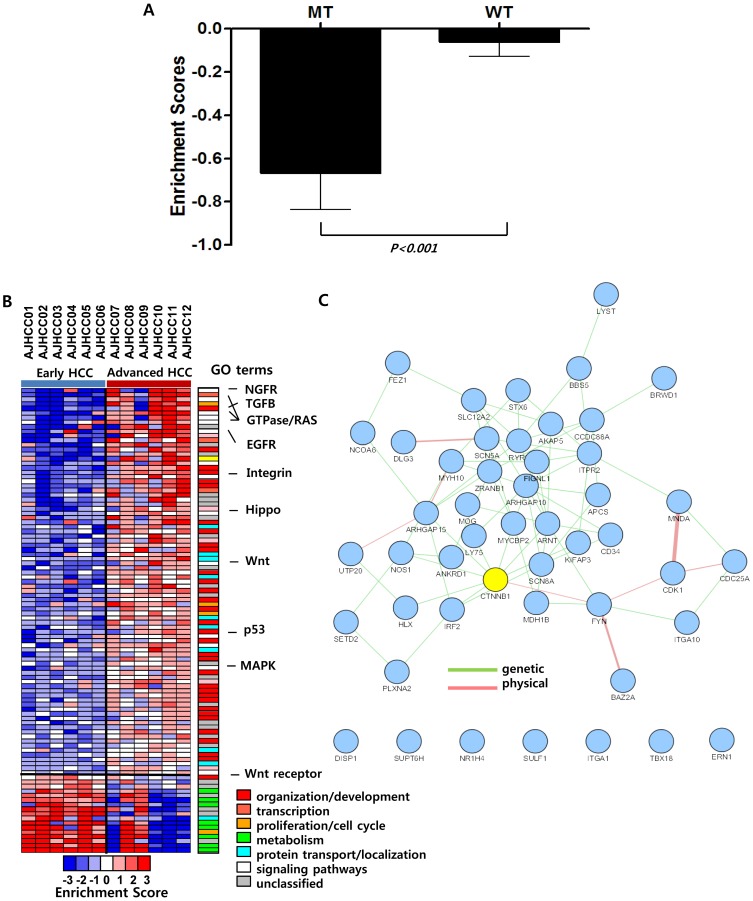
Differential spectrum of tumor-specific variations are related with the transcriptional deregulation of the early and advanced HCC. **A.** The barplot shows the average enrichment scores of the gene expressions in the GO terms in each patient. The enrichment scores of the gene sets are calculated as described in Method. The GO terms of the genes with mutations and the ones without mutations are indicated as mutated type (MT) and wild type (WT), respectively. **B.** The heatmap shows the differentially enriched functions of mutated genes with deregulated expression. The categories with similar functions are indicated as a barplot of different colors (*right bar*). **C.** Network view of the 48 mutated genes with differential expression between the early and advanced HCC subgroups. The *CTNNB1* gene harbored the largest interaction partners indicated with yellow color.

To evaluate the relationships between the mutations and transcriptional deregulations in HCC, we performed gene expression profiling with the same patients' samples. To address the functional association, we assigned the mutated genes and the gene expressions to the gene ontology (GO) terms. To predict the functional alteration by gene mutations, a total of 682 GO terms were selected which had at least one mutations in our dataset. Then, the functional enrichment of gene expressions for each patient was calculated as described in Methods. Strikingly, we observed that the expression levels of the mutated genes were significantly lower than those of the wild-type genes (Two-tailed Student's T-test, p = 0.0006) (**Fig. 3A**). This may indicate the gene mutations might be the inactivation mutations rather than the activation mutations.

Next, we compared the differential gene expressions between early and advanced HCC subgroups. Of the 682 GO terms, a total of 102 GO terms with the 48 mutated genes were identified which had different enrichment scores (*P*<0.05) with greater than two-fold difference between the subgroups. Interestingly, the advanced HCC were significantly enriched with aggressiveness-related functions including cytoskeleton organization, development, proliferation, and cell migration-related genes, while the early HCCs were enriched with metabolism-related genes (**Fig. 3B**). Cancer-associated pathways including NGFR, TGFB, Hippo, and Wnt were also deregulated between the HCC subgroups, implying that the differential functions were involved in HCC progression. This result suggests that the different mutation profiles with concomitant transcriptional deregulation may contribute to the heterogeneous progression of the early and the advanced HCCs.

In addition, we sought to identify key regulators among the 48 mutated genes which are likely to associate with the subgroup-dependent mutations and concomitant gene expression. By applying GeneMANIA software implemented in Cytoscape plugin [33], we constructed a network using the genetic and physical interactions among the 48 mutated genes. The mutated genes were found to be closely linked together suggesting that the mutations might be actionable in the differential development of HCCs (**Fig. 3C**). Particularly, *CTNNB1* gene, which is well known to play critical roles in HCC development and progression, had the largest number of interaction partners in the network. This may indicate that the mutation-derived disruption of *CTNNB1* and its pathways might play important roles in the progression of the HCC subgroups. This observation is concordant with the previous results that the HCCs with *CTNNB1* mutations were early staged and had a differentiated phenotype [34]. It has also been known that the *CTNNB1* mutations are associated with better prognosis of HCC [35], while the activation of Wnt pathways is correlated with poor prognosis [36].

## Discussion

Although the NGS technologies could provide large scale profiles of genome-wide mutations in cancers during the past years, there are limitations in the assessment of functional implications of the mutation profile. Somatic mutations of cancers showed marked individual variations, therefore, it might be difficult to find key regulators with frequent mutations by analyzing the small sample sized data. Moreover, the action of the mutants can be diverse according to their base positions. The mutations from different base positions of the same gene can lead to different gene functions such as inactivation and activation mutations. This might be resulted from their effects on the physico-chemical properties and structures of wild-type proteins. Moreover, intra-tumoral heterogeneity may also contribute to false-negative calls in the tumors especially with low copy variations [23]. Besides, low sensitivity and specificity of the current high-throughput sequencing technologies may preclude the acquisition of accurate mutation profiles [4]. In addition, the erroneous mutation calls can be derived from base calling algorithms. For example, the genes encoding extremely large proteins were found to be frequently mutated, demanding further development of more accurate and significant base calling algorithms [37]. Considering these limitations of the mutation profiling studies, large sample and multiple assays might be necessarily required to get accurate information for predicting functional and clinical outcomes. Validation of the individual mutations with different methods might be required to overcome such limitations of NGS technology [38]. Notwithstanding such limitations of the mutation profiling studies, gene set-based analysis of the mutations such as gene set enrichment analysis could have successfully demonstrated their functional significance in cancers. For example, frequent mutations of chromatin regulators [8], Wnt/beta catenin, and Jak/STAT pathways [10] have been noticed in HCC, although the individual variants have low mutation rates. These results suggest that the assessment of the enriched mutations of a certain gene set rather than a single gene mutation is a useful approach to predict the functional role of the mutation profiles on cancer progression. Of course, these mutations could be prioritized targets for cancer treatment.

In this study, we performed a mutation profiling of HCC by whole-exome sequencing analysis. Comparing the mutations and gene expression profiles between the early and the advanced HCC, we could observe the tumor grade-specific mutation patterns and their associations with the gene expression levels. Considering the high rates of false mutation calls of whole exome-seq data as described above, we applied stringent algorithms for variant calling.

In addition, our mutation set-based analysis could reveal new insights on the somatic mutations of HCC. By applying comparative and integrative approaches, we could reveal regional and functional patterns of the variants. Remarkably, we found that the chromosome 1q had frequent tumor-specific mutations particularly in the advanced HCC. Previously, genomic alterations at chromosome 1q have been noticed to have functional and clinical significance in the HCC progression. For example, recurrent copy number gains at 1q were observed to associate with the aggressive cancer behavior [39], [40]. This may indicate that the frequent genomic aberrations and instability at 1q including genome copy numbers and mutations contribute to the aggressive phenotype of HCC.

Functional enrichment analysis revealed the differential enrichment of mutations between the early and the advanced HCCs. The cytoskeleton and protein transport-related genes were enriched in the early HCC, while the cell migration and ion transport-related genes were enriched in the advanced HCC. These analyses successfully demonstrated that the differential mutations between the early and advanced HCC were well reflected at transcriptional level. Particularly, the mutations of ion-transport genes were largely located at chromosome 1q, which might be associated with the enriched mutations of 1q in the advanced HCC. Furthermore, it is interesting to find that the cancer-associated pathways including NGFR, TGFB, Hippo, and Wnt had the differential mutations and expressions between the HCC subgroups (see **Fig. 3B**). The integrative network analysis could reveal that the 48 gene mutations including *CTNNB1*, which might have pivotal roles in the heterogeneous progression of HCC subgroup.

In conclusion, our integrative analysis using exome-seq and gene expression profiles could provide genome-wide landscape of mutations in the early and the advanced HCC. By demonstrating the effects of the mutation sets on the transcriptional level, we could identify the potentially actionable tumor-specific mutations, which could be personalized and therapeutic targets for HCC diagnostics and treatment.

## Supporting Information

S1 Figure **The number of mutations in each patient.** The number of tumor-specific and non-tumor-specific mutations are plotted.
(PDF)

S2 Figure **Mutation spectrum of non-tumor-specific mutations.** Distribution of somatic non-tumor-specific variants in HCC. B. The heatmap indicate the enrichment scores of non-tumor-specific variants which calculated as the odds ratios of the numbers of variants in each chromosome arm against those of outside the chromosome arm in each patient. The enrichment score less than 1 was truncated to zero.
(PDF)

S3 Figure **Probability of the flanking sequences of the tumor-specific mutation sites.** Positional probability of the flanking sequences of the each of the reference and the mutated nucleotide base of the tumor-specific mutations are plotted by using Weblogo software.
(PDF)

S4 Figure **Tumor-specific mutations at gene level were enriched with cell cycle-related genes.** **A**. Ben-diagram show the number the tumor-specific and the non-tumor-specific mutations at gene level. **B.** The tumor specific mutated genes in cell-cycle pathway from KEGG are indicated by red color. C. Of the genes harboring the tumor-specific and the non-tumor-specific mutations, cell cycle-related genes are shown.
(PDF)

S1 Table **Clinical and pathological features of HCC samples.**
(DOCX)

S2 Table **List of tumor-specific and non-tumor-specific variations in the early and the advanced HCC.**
(DOCX)

S3 Table **Functional enrichment of non-tumoral specific variants.**
(DOCX)
