# Supplementary material for: Profiling of Exome Mutations Associated with Progression of HBV-Related Hepatocellular Carcinoma
Source: PLoS One. 2014 Dec 18;9(12):e115152. doi: 10.1371/journal.pone.0115152 (PMC4270755; doi:10.1371/journal.pone.0115152)
Supplement: S1 Figure — The number of mutations in each patient. The number of tumor-specific and non-tumor-specific mutations are plotted. (PDF) [file pone.0115152.s001.pdf]

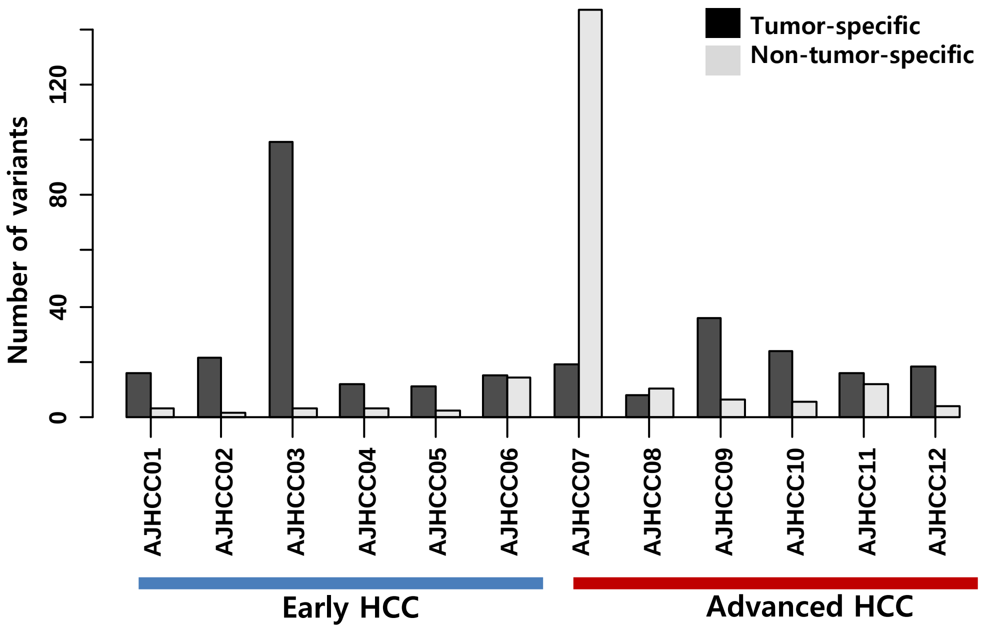

Figure. S1. The number of mutations in each patient
The number of tumor-specific and non-tumor-specific mutations are plotted.
