# Supplementary material for: Profiling of Exome Mutations Associated with Progression of HBV-Related Hepatocellular Carcinoma
Source: PLoS One. 2014 Dec 18;9(12):e115152. doi: 10.1371/journal.pone.0115152 (PMC4270755; doi:10.1371/journal.pone.0115152)
Supplement: S2 Figure — Mutation spectrum of non-tumor-specific mutations. Distribution of somatic non-tumor-specific variants in HCC. B. The heatmap indicate the enrichment scores of non-tumor-specific variants which calculated as the odds ratios of the numbers of variants in each chromosome arm against those of outside the chromosome arm in each patient. The enrichment score less than 1 was truncated to zero. (PDF) [file pone.0115152.s002.pdf]

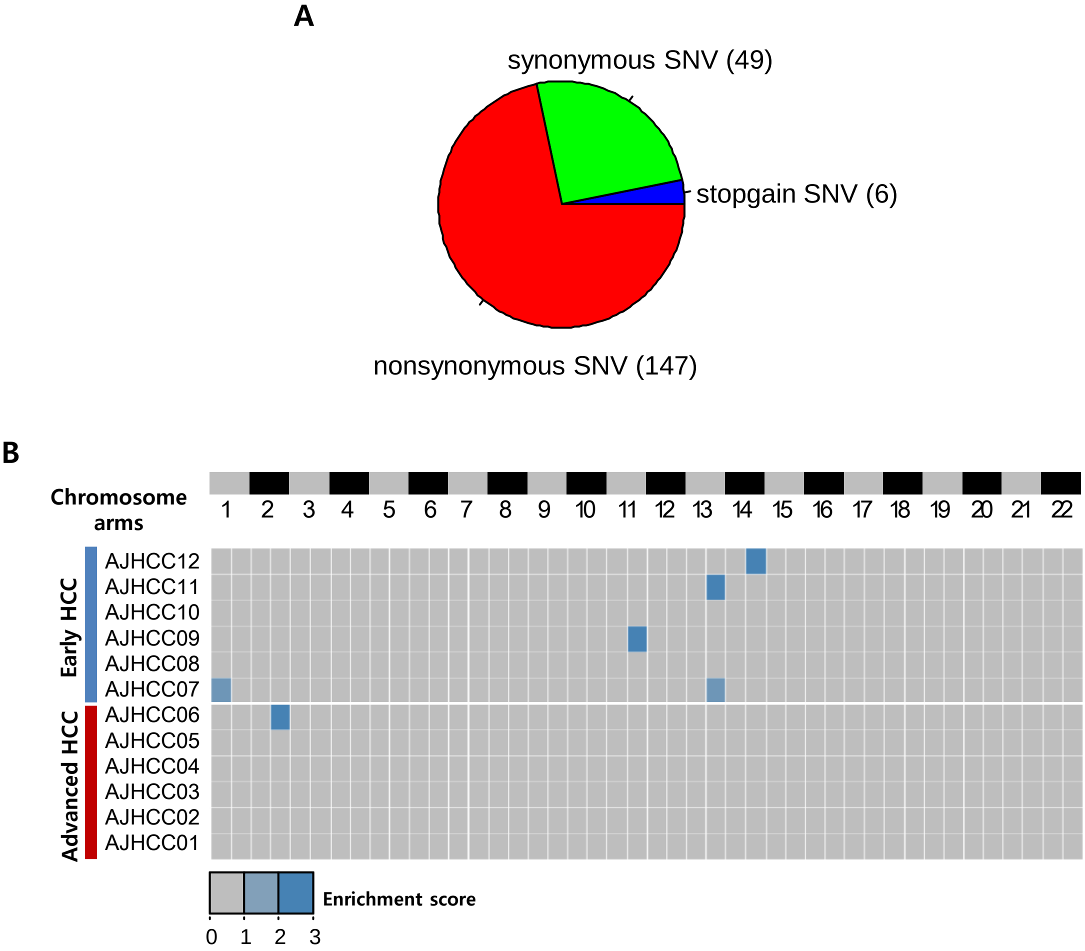

Figure. S2. Mutation spectrum of non-tumor-specific mutations
A. Distribution of somatic non-tumor-specific variants in HCC. **B**. The heatmap indicate the enrichment scores of non-tumor-specific variants which calculated as the odds ratios of the numbers of variants in each chromosome arm against those of outside the chromosome arm in each patient. The enrichment score less than 1 was truncated to zero.
