# Supplementary material for: Profiling of Exome Mutations Associated with Progression of HBV-Related Hepatocellular Carcinoma
Source: PLoS One. 2014 Dec 18;9(12):e115152. doi: 10.1371/journal.pone.0115152 (PMC4270755; doi:10.1371/journal.pone.0115152)
Supplement: S3 Figure — Probability of the flanking sequences of the tumor-specific mutation sites. Positional probability of the flanking sequences of the each of the reference and the mutated nucleotide base of the tumor-specific mutations are plotted by using Weblogo software. (PDF) [file pone.0115152.s003.pdf]

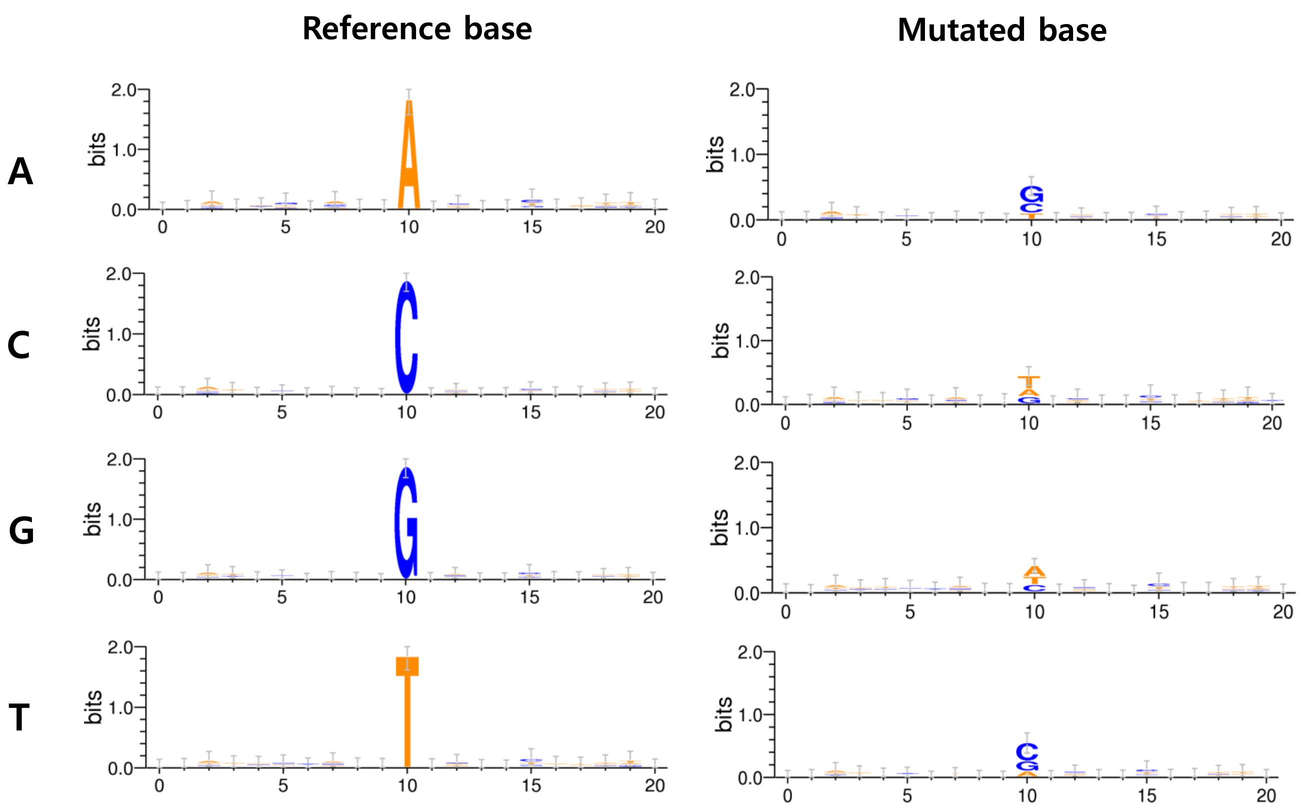

Figure. S3. Probability of the flanking sequences of the tumor-specific mutation sites
Positional probability of the flanking sequences of the each of the reference and the mutated nucleotide base of the tumor-specific mutations are plotted by using Weblogo software.
