# Supplementary material for: Profiling of Exome Mutations Associated with Progression of HBV-Related Hepatocellular Carcinoma
Source: PLoS One. 2014 Dec 18;9(12):e115152. doi: 10.1371/journal.pone.0115152 (PMC4270755; doi:10.1371/journal.pone.0115152)
Supplement: S4 Figure — Tumor-specific mutations at gene level were enriched with cell cycle-related genes. A. Ben-diagram show the number the tumor-specific and the non-tumor-specific mutations at gene level. B. The tumor specific mutated genes in cell-cycle pathway from KEGG are indicated by red color. C. Of the genes harboring the tumor-specific and the non-tumor-specific mutations, cell cycle-related genes are shown. (PDF) [file pone.0115152.s004.pdf]

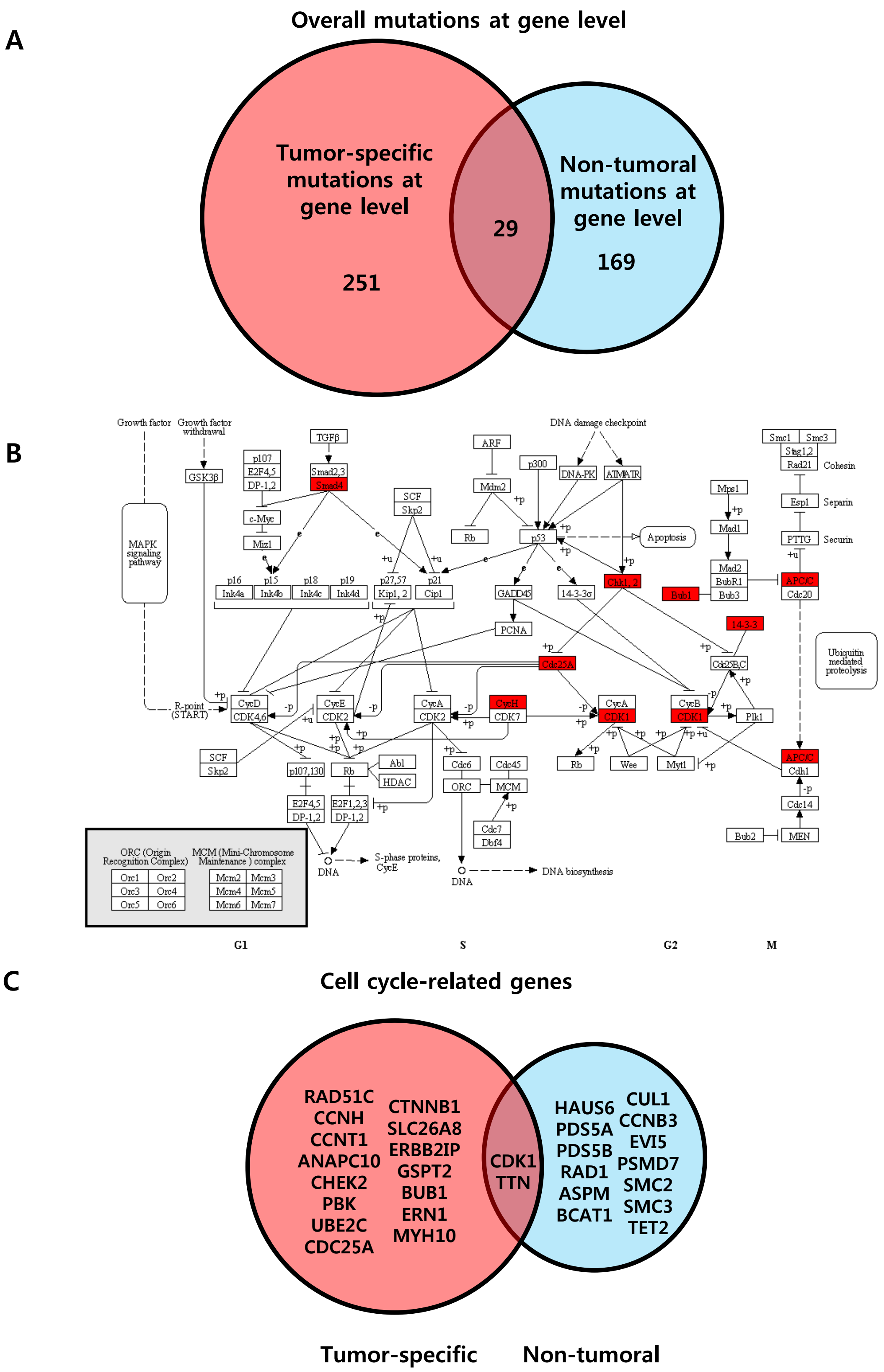

Figure. S4. Tumor-specific mutations at gene level were enriched with cell cycle-related genes
A. Ben-diagram show the number the tumor-specific and the non-tumor-specific mutations at gene level. **B.** The tumor specific mutated genes in cell-cycle pathway from KEGG are indicated by red color. **C**. Of the genes harboring the tumor-specific and the non-tumor-specific mutations, cell cycle-related genes are shown.
