# Supplementary material for: Profiling of Exome Mutations Associated with Progression of HBV-Related Hepatocellular Carcinoma
Source: PLoS One. 2014 Dec 18;9(12):e115152. doi: 10.1371/journal.pone.0115152 (PMC4270755; doi:10.1371/journal.pone.0115152)
Supplement: S1 Table — Clinical and pathological features of HCC samples. (DOCX) [file pone.0115152.s005.docx]

## Table S1. Clinical and pathological features of HCC samples

| Features | Early HCC | Advanced HCC |
| --- | --- | --- |
| **Sex (Male/Female)** | 4/2 | 4/2 |
| **Age (mean ± S.D)** | 53.5±11.97 | 52.1±7.67 |
| **Tumor Size (mean ± S.D)** | 4.133±1.21 | 7.11±6.16 |
| **Tumor type (single/multiple)** | 6/0 | 1/5 |
| **micro invasion (-/+)** | 5/1 | 4/2 |
| **macro invasion(-/+)** | 6/0 | 3/3 |
| **UICC (T1,T2/T3,T4)** | 6/0 | 0/6 |
| **Edmonson Grade (I,II/III,IV)** | 6/0 | 0/6 |
| **AFP (>400/<400)** | 1/5 | 3/3 |
| **Child Score (A/B)** | 5/1 | 6/0 |
