# Supplementary material for: Profiling of Exome Mutations Associated with Progression of HBV-Related Hepatocellular Carcinoma
Source: PLoS One. 2014 Dec 18;9(12):e115152. doi: 10.1371/journal.pone.0115152 (PMC4270755; doi:10.1371/journal.pone.0115152)
Supplement: S2 Table — List of tumor-specific and non-tumor-specific variations in the early and the advanced HCC. (DOCX) [file pone.0115152.s006.docx]

## Table S2. List of tumor-specific and non-tumor-specific variations in the early and the advanced HCC

| **Cytoband** | **Position** | **Variations** | **Gene** | **Exonic Function** |
| --- | --- | --- | --- | --- |
| **Tumor-specific variations** | | | | |
| 1p36.32 | 2318900 | C>T | MORN1 | nonsynonymous |
| 1p36.11 | 26098153 | G>A | MAN1C1 | nonsynonymous |
| 1p32.1 | 59127098 | T>A | MYSM1 | nonsynonymous |
| 1p22.3 | 86960018 | T>C | CLCA1 | nonsynonymous |
| 1p21.2 | 100154771 | C>A | PALMD | nonsynonymous |
| 1p13.3 | 107867382 | C>T | NTNG1 | nonsynonymous |
| 1p13.3 | 110923732 | G>C | SLC16A4 | nonsynonymous |
| 1q21.1 | 145528605 | C>G | ITGA10 | nonsynonymous |
| 1q21.2 | 150053510 | C>G | VPS45 | nonsynonymous |
| 1q21.3 | 150807059 | A>G | ARNT | nonsynonymous |
| 1q21.3 | 152057477 | A>C | TCHHL1 | nonsynonymous |
| 1q21.3 | 152059046 | T>G | TCHHL1 | nonsynonymous |
| 1q21.3 | 152128542 | G>T | RPTN | nonsynonymous |
| 1q21.3 | 153043257 | G>A | SPRR2B | nonsynonymous |
| 1q22 | 155698872 | A>G | DAP3 | nonsynonymous |
| 1q23.1 | 158817689 | A>C | MNDA | nonsynonymous |
| 1q23.1 | 158817698 | T>G | MNDA | nonsynonymous |
| 1q23.2 | 159557717 | C>A | APCS | nonsynonymous |
| 1q23.3 | 161198850 | A>T | TOMM40L | stopgain |
| 1q23.3 | 165377488 | G>C | RXRG | nonsynonymous |
| 1q24.2 | 169267935 | T>C | NME7 | nonsynonymous |
| 1q24.2 | 169930306 | T>C | KIFAP3 | nonsynonymous |
| 1q25.2 | 179076856 | C>A | ABL2 | nonsynonymous |
| 1q25.2 | 179975644 | C>A | CEP350 | synonymous |
| 1q25.3 | 180953864 | G>A | STX6 | nonsynonymous |
| 1q25.3 | 181741312 | G>A | CACNA1E | nonsynonymous |
| 1q25.3 | 182555622 | A>G | RNASEL | nonsynonymous |
| 1q31.1 | 185931689 | T>C | HMCN1 | nonsynonymous |
| 1q31.1 | 185931778 | G>A | HMCN1 | nonsynonymous |
| 1q32.1 | 200817319 | C>A | CAMSAP2 | nonsynonymous |
| 1q32.2 | 208072525 | A>C | CD34 | stopgain |
| 1q32.2 | 208269469 | G>A | PLXNA2 | synonymous |
| 1q32.2 | 208272311 | G>C | PLXNA2 | nonsynonymous |
| 1q32.2 | 208272313 | A>C | PLXNA2 | nonsynonymous |
| 1q32.3 | 213436143 | G>C | RPS6KC1 | nonsynonymous |
| 1q41 | 221055507 | T>G | HLX | synonymous |
| 1q41 | 222838904 | A>C | MIA3 | synonymous |
| 1q41 | 223175813 | C>G | DISP1 | nonsynonymous |
| 1q42.12 | 225446846 | G>C | DNAH14 | nonsynonymous |
| 1q42.2 | 232942854 | G>A | KIAA1383 | synonymous |
| 1q42.3 | 235969192 | C>T | LYST | nonsynonymous |
| 1q43 | 237889582 | C>T | RYR2 | nonsynonymous |
| 1q44 | 244735743 | C>T | C1orf101 | nonsynonymous |
| 1q44 | 248487109 | T>A | OR2M7 | synonymous |
| 2p23.2 | 29416173 | C>T | ALK | nonsynonymous |
| 2p22.3 | 33813450 | A>G | FAM98A | synonymous |
| 2p22.1 | 39996936 | C>G | THUMPD2 | nonsynonymous |
| 2p21 | 44573443 | T>G | PREPL | synonymous |
| 2p16.1 | 55563867 | C>G | CCDC88A | nonsynonymous |
| 2p13.2 | 73478374 | T>G | CCT7 | synonymous |
| 2p12 | 77746143 | C>T | LRRTM4 | synonymous |
| 2p11.2 | 88327635 | G>T | KRCC1 | nonsynonymous |
| 2q12.2 | 107446591 | G>C | ST6GAL2 | nonsynonymous |
| 2q13 | 111431750 | T>A | BUB1 | synonymous |
| 2q13 | 113736881 | C>T | IL36G | stopgain |
| 2q22.1 | 141032064 | C>A | LRP1B | nonsynonymous |
| 2q22.3 | 144461049 | G>T | ARHGAP15 | synonymous |
| 2q24.2 | 160690622 | G>A | LY75 | synonymous |
| 2q24.3 | 168106939 | A>G | XIRP2 | nonsynonymous |
| 2q31.1 | 170338767 | G>A | BBS5 | nonsynonymous |
| 2q31.2 | 179550042 | T>C | TTN | nonsynonymous |
| 2q32.1 | 183595807 | C>G | DNAJC10 | nonsynonymous |
| 2q32.2 | 191382263 | T>C | TMEM194B | nonsynonymous |
| 2q33.1 | 198266711 | T>G | SF3B1 | nonsynonymous |
| 2q33.1 | 201534325 | G>T | AOX1 | nonsynonymous |
| 2q33.3 | 207619856 | G>C | MDH1B | nonsynonymous |
| 2q34 | 210685150 | A>T | UNC80 | nonsynonymous |
| 2q34 | 210707151 | G>C | UNC80 | nonsynonymous |
| 2q35 | 217055046 | G>A | XRCC5 | nonsynonymous |
| 2q37.1 | 234438147 | G>A | USP40 | nonsynonymous |
| 2q37.1 | 234839375 | T>C | TRPM8 | synonymous |
| 2q37.1 | 234969043 | G>A | SPP2 | nonsynonymous |
| 3p25.1 | 15718550 | C>A | ANKRD28 | nonsynonymous |
| 3p22.2 | 37107365 | T>C | LRRFIP2 | synonymous |
| 3p22.2 | 37366903 | A>G | GOLGA4 | nonsynonymous |
| 3p22.2 | 38539120 | G>T | EXOG | nonsynonymous |
| 3p22.2 | 38592804 | C>T | SCN5A | nonsynonymous |
| 3p22.1 | 41274911 | T>G | CTNNB1 | nonsynonymous |
| 3p21.31 | 44441983 | A>G | TCAIM | nonsynonymous |
| 3p21.31 | 46245454 | C>A | CCR1 | nonsynonymous |
| 3p21.31 | 46414729 | C>T | CCR5 | synonymous |
| 3p21.31 | 47127742 | C>T | SETD2 | stopgain |
| 3p21.31 | 48222313 | C>T | CDC25A | synonymous |
| 3p21.2 | 51394463 | T>C | DOCK3 | nonsynonymous |
| 3p21.1 | 53139768 | A>G | RFT1 | nonsynonymous |
| 3p14.3 | 55021756 | T>A | CACNA2D3 | stopgain |
| 3q11.2 | 97806182 | C>A | OR5AC2 | nonsynonymous |
| 3q12.1 | 99568437 | T>G | FILIP1L | nonsynonymous |
| 3q13.2 | 112262936 | C>T | ATG3 | nonsynonymous |
| 3q13.2 | 112289466 | T>A | SLC35A5 | synonymous |
| 3q13.2 | 113304110 | T>A | SIDT1 | nonsynonymous |
| 3q21.3 | 129152940 | G>T | MBD4 | nonsynonymous |
| 3q22.1 | 130119896 | T>A | COL6A5 | nonsynonymous |
| 3q22.1 | 133558421 | G>A | RAB6B | synonymous |
| 3q22.3 | 135980834 | G>C | PCCB | nonsynonymous |
| 3q26.1 | 164735791 | G>A | SI | nonsynonymous |
| 3q26.33 | 179137236 | T>A | GNB4 | nonsynonymous |
| 3q28 | 190362135 | G>A | IL1RAP | nonsynonymous |
| 3q29 | 194373752 | T>A | LSG1 | synonymous |
| 4p15.2 | 26744213 | G>T | TBC1D19 | synonymous |
| 4p14 | 37447746 | G>C | KIAA1239 | nonsynonymous |
| 4p14 | 40133492 | T>C | N4BP2 | synonymous |
| 4p12 | 47901468 | C>A | NFXL1 | nonsynonymous |
| 4q12 | 57319806 | C>G | PAICS | synonymous |
| 4q22.1 | 90034795 | A>T | TIGD2 | nonsynonymous |
| 4q24 | 106473935 | G>A | ARHGEF38 | nonsynonymous |
| 4q27 | 123664203 | C>T | BBS12 | nonsynonymous |
| 4q31.21 | 145916659 | G>A | ANAPC10 | synonymous |
| 4q31.23 | 148743964 | C>A | ARHGAP10 | synonymous |
| 4q31.3 | 151727485 | G>A | LRBA | nonsynonymous |
| 4q35.1 | 185340691 | T>A | IRF2 | nonsynonymous |
| 5p15.2 | 11018155 | C>A | CTNND2 | synonymous |
| 5p15.1 | 16668446 | G>T | MYO10 | synonymous |
| 5p13.3 | 31421419 | A>G | DROSHA | nonsynonymous |
| 5q11.2 | 52145314 | A>G | ITGA1 | synonymous |
| 5q11.2 | 54585173 | C>A | DHX29 | nonsynonymous |
| 5q12.3 | 63510124 | C>G | RNF180 | nonsynonymous |
| 5q12.3 | 64625291 | T>C | ADAMTS6 | synonymous |
| 5q12.3 | 65374262 | C>A | ERBB2IP | stopgain |
| 5q14.1 | 79025224 | C>A | CMYA5 | nonsynonymous |
| 5q14.1 | 79837622 | C>A | FAM151B | nonsynonymous |
| 5q14.3 | 86705168 | T>A | CCNH | nonsynonymous |
| 5q14.3 | 89977150 | C>T | GPR98 | nonsynonymous |
| 5q15 | 93985196 | C>T | ANKRD32 | nonsynonymous |
| 5q22.2 | 111600624 | C>T | EPB41L4A | nonsynonymous |
| 5q23.3 | 127450348 | A>G | SLC12A2 | nonsynonymous |
| 5q31.2 | 138652744 | G>T | MATR3 | nonsynonymous |
| 5q31.3 | 140515971 | G>T | PCDHB5 | nonsynonymous |
| 5q31.3 | 140572512 | C>T | PCDHB10 | synonymous |
| 5q31.3 | 140580504 | C>A | PCDHB11 | nonsynonymous |
| 5q33.2 | 153382488 | G>T | FAM114A2 | synonymous |
| 5q33.2 | 154396360 | C>T | KIF4B | stopgain |
| 5q35.1 | 170610337 | C>T | RANBP17 | synonymous |
| 6p22.2 | 26158684 | A>G | HIST1H2BD | nonsynonymous |
| 6p22.2 | 26463611 | A>G | BTN2A1 | synonymous |
| 6p22.1 | 27833427 | G>C | HIST1H2AL | nonsynonymous |
| 6p22.1 | 29080073 | A>T | OR2J3 | nonsynonymous |
| 6p22.1 | 29635681 | A>C | MOG | nonsynonymous |
| 6p21.31 | 34846438 | C>T | TAF11 | nonsynonymous |
| 6p21.31 | 35911681 | A>C | SLC26A8 | nonsynonymous |
| 6p21.1 | 44217777 | T>G | HSP90AB1 | synonymous |
| 6p12.2 | 52849285 | T>C | GSTA4 | nonsynonymous |
| 6q14.1 | 83855356 | A>C | DOPEY1 | synonymous |
| 6q14.2 | 84055984 | G>A | ME1 | nonsynonymous |
| 6q14.3 | 85466496 | A>C | TBX18 | nonsynonymous |
| 6q21 | 110567301 | A>T | METTL24 | nonsynonymous |
| 6q21 | 112015658 | A>T | FYN | nonsynonymous |
| 6q22.1 | 116784695 | C>G | FAM26F | nonsynonymous |
| 6q22.1 | 117130644 | C>G | GPRC6A | nonsynonymous |
| 6q22.31 | 118832472 | C>T | CEP85L | nonsynonymous |
| 6q22.31 | 118887190 | T>C | CEP85L | synonymous |
| 6q22.33 | 127797390 | A>C | SOGA3 | nonsynonymous |
| 6q23.2 | 131926514 | T>A | MED23 | synonymous |
| 6q23.2 | 132785206 | C>T | STX7 | nonsynonymous |
| 6q23.2 | 133138165 | C>T | RPS12 | nonsynonymous |
| 6q25.1 | 149340313 | C>T | UST | synonymous |
| 6q25.1 | 151789894 | G>A | C6orf211 | nonsynonymous |
| 7p14.1 | 37924829 | G>A | NME8 | nonsynonymous |
| 7p12.1 | 50513686 | G>T | FIGNL1 | nonsynonymous |
| 7p11.2 | 55498929 | C>G | LANCL2 | synonymous |
| 7q21.11 | 82544761 | C>G | PCLO | nonsynonymous |
| 7q21.11 | 82581956 | G>A | PCLO | synonymous |
| 7q21.13 | 89915636 | G>A | C7orf63 | nonsynonymous |
| 7q21.2 | 92762824 | C>T | SAMD9L | nonsynonymous |
| 7q21.3 | 94043209 | G>T | COL1A2 | nonsynonymous |
| 7q31.32 | 122757532 | A>T | SLC13A1 | nonsynonymous |
| 8p21.1 | 27679859 | A>G | PBK | synonymous |
| 8p11.21 | 41552749 | T>C | ANK1 | nonsynonymous |
| 8q13.2 | 68084692 | G>A | CSPP1 | nonsynonymous |
| 8q13.2 | 70498629 | C>G | SULF1 | nonsynonymous |
| 8q13.3 | 71510200 | A>C | TRAM1 | nonsynonymous |
| 8q21.11 | 74235233 | C>A | RDH10 | nonsynonymous |
| 8q22.3 | 101727772 | T>C | PABPC1 | synonymous |
| 8q23.1 | 110100284 | C>A | TRHR | stopgain |
| 8q24.13 | 126369490 | G>A | NSMCE2 | nonsynonymous |
| 8q24.13 | 126369965 | G>A | NSMCE2 | synonymous |
| 8q24.21 | 130859110 | C>G | FAM49B | nonsynonymous |
| 8q24.22 | 133622480 | C>T | LRRC6 | nonsynonymous |
| 8q24.22 | 134128945 | A>T | TG | nonsynonymous |
| 8q24.3 | 141445232 | C>G | TRAPPC9 | nonsynonymous |
| 8q24.3 | 145278040 | C>T | HEATR7A | synonymous |
| 9p21.2 | 26905862 | G>C | PLAA | nonsynonymous |
| 9p21.2 | 27016979 | A>G | IFT74 | synonymous |
| 9p21.2 | 27016980 | G>A | IFT74 | nonsynonymous |
| 9q21.2 | 79954569 | G>A | VPS13A | synonymous |
| 9q32 | 115760521 | A>G | ZNF883 | nonsynonymous |
| 9q34.13 | 134814858 | A>G | MED27 | synonymous |
| 10p13 | 17061867 | C>T | CUBN | nonsynonymous |
| 10q11.21 | 44052203 | T>A | ZNF239 | nonsynonymous |
| 10q11.23 | 50034834 | G>A | WDFY4 | nonsynonymous |
| 10q21.1 | 54048543 | T>G | PRKG1 | nonsynonymous |
| 10q21.2 | 62544513 | G>T | CDK1 | nonsynonymous |
| 10q21.3 | 69692484 | G>A | HERC4 | nonsynonymous |
| 10q21.3 | 70196869 | A>C | DNA2 | synonymous |
| 10q23.31 | 92678921 | A>T | ANKRD1 | synonymous |
| 10q24.1 | 97620289 | G>T | ENTPD1 | stopgain |
| 10q26.13 | 126631773 | T>G | ZRANB1 | synonymous |
| 10q26.2 | 128974295 | C>T | FAM196A | nonsynonymous |
| 11p15.4 | 4615936 | T>C | OR52I1 | nonsynonymous |
| 11p15.1 | 18637478 | T>G | SPTY2D1 | nonsynonymous |
| 11q12.1 | 56020253 | C>A | OR5T3 | nonsynonymous |
| 11q12.2 | 60105342 | A>G | MS4A6E | nonsynonymous |
| 11q22.3 | 103049922 | T>C | DYNC2H1 | nonsynonymous |
| 11q23.1 | 111622970 | A>G | PPP2R1B | synonymous |
| 11q23.2 | 113674583 | T>C | USP28 | nonsynonymous |
| 11q23.3 | 118452130 | A>C | ARCN1 | nonsynonymous |
| 11q24.2 | 125333434 | C>T | FEZ1 | synonymous |
| 11q24.3 | 129748385 | C>T | NFRKB | nonsynonymous |
| 11q25 | 134128490 | G>A | ACAD8 | synonymous |
| 12p13.2 | 10133310 | A>C | CLEC12A | nonsynonymous |
| 12p12.2 | 20870095 | C>T | SLCO1C1 | nonsynonymous |
| 12p11.23 | 26564299 | T>C | ITPR2 | synonymous |
| 12q12 | 43925912 | G>T | ADAMTS20 | nonsynonymous |
| 12q13.11 | 49089571 | T>G | CCNT1 | nonsynonymous |
| 12q13.12 | 50500201 | C>T | GPD1 | nonsynonymous |
| 12q13.13 | 52188220 | G>A | SCN8A | nonsynonymous |
| 12q13.13 | 52188367 | C>G | SCN8A | stopgain |
| 12q13.3 | 57005382 | C>A | BAZ2A | stopgain |
| 12q13.3 | 57883106 | A>G | MARS | nonsynonymous |
| 12q14.2 | 64818469 | G>A | XPOT | nonsynonymous |
| 12q15 | 68051240 | G>T | DYRK2 | nonsynonymous |
| 12q23.1 | 100904632 | T>G | NR1H4 | synonymous |
| 12q23.2 | 101767184 | A>G | UTP20 | nonsynonymous |
| 12q23.2 | 102067380 | C>G | MYBPC1 | stopgain |
| 12q24.11 | 110893460 | T>C | GPN3 | synonymous |
| 12q24.22 | 117664515 | T>C | NOS1 | nonsynonymous |
| 12q24.31 | 124236838 | C>A | ATP6V0A2 | stopgain |
| 13q12.11 | 22095384 | C>T | EFHA1 | synonymous |
| 13q22.2 | 76287354 | C>G | LMO7 | nonsynonymous |
| 13q22.3 | 77673117 | T>G | MYCBP2 | nonsynonymous |
| 13q34 | 111276615 | G>A | CARKD | nonsynonymous |
| 13q34 | 113200052 | G>A | TUBGCP3 | synonymous |
| 13q34 | 113732714 | C>T | MCF2L | synonymous |
| 14q12 | 31358852 | G>T | COCH | nonsynonymous |
| 14q13.2 | 36103797 | A>G | RALGAPA1 | nonsynonymous |
| 14q22.1 | 52899305 | G>A | TXNDC16 | nonsynonymous |
| 14q23.1 | 59757965 | G>A | DAAM1 | nonsynonymous |
| 14q23.2 | 62462748 | C>T | SYT16 | nonsynonymous |
| 14q23.3 | 64935947 | G>A | AKAP5 | nonsynonymous |
| 14q32.33 | 105407334 | A>G | AHNAK2 | synonymous |
| 15q15.1 | 41108174 | G>A | PPP1R14D | nonsynonymous |
| 15q15.1 | 42602928 | A>G | GANC | nonsynonymous |
| 15q15.2 | 43281052 | G>A | UBR1 | nonsynonymous |
| 15q21.2 | 50929756 | A>T | TRPM7 | nonsynonymous |
| 15q21.2 | 51766637 | C>A | DMXL2 | nonsynonymous |
| 15q22.2 | 62214646 | T>C | VPS13C | nonsynonymous |
| 15q22.31 | 65748581 | G>A | DPP8 | synonymous |
| 15q26.3 | 101717871 | C>A | CHSY1 | nonsynonymous |
| 16q12.2 | 55905615 | C>T | CES5A | synonymous |
| 16q21 | 58566266 | C>T | CNOT1 | nonsynonymous |
| 16q23.3 | 84199455 | G>C | DNAAF1 | nonsynonymous |
| 17p13.3 | 1264449 | C>A | YWHAE | nonsynonymous |
| 17p13.1 | 8526497 | T>G | MYH10 | nonsynonymous |
| 17q11.2 | 27001584 | G>A | SUPT6H | nonsynonymous |
| 17q12 | 32961994 | T>G | TMEM132E | nonsynonymous |
| 17q21.33 | 48456013 | T>C | EME1 | nonsynonymous |
| 17q22 | 56774108 | T>G | RAD51C | synonymous |
| 17q23.3 | 62157038 | T>C | ERN1 | nonsynonymous |
| 17q24.3 | 67264088 | T>C | ABCA5 | nonsynonymous |
| 18q12.1 | 31263418 | A>T | ASXL3 | synonymous |
| 18q12.1 | 31318857 | G>T | ASXL3 | nonsynonymous |
| 18q21.2 | 48591931 | G>C | SMAD4 | nonsynonymous |
| 18q22.1 | 64176319 | T>G | CDH19 | nonsynonymous |
| 18q22.2 | 67406229 | A>C | DOK6 | nonsynonymous |
| 18q22.3 | 71920894 | C>A | CYB5A | nonsynonymous |
| 19p13.2 | 9677375 | A>G | ZNF121 | synonymous |
| 19p13.2 | 12256835 | C>T | ZNF625 | synonymous |
| 19p13.11 | 19822632 | A>C | ZNF14 | synonymous |
| 19q13.2 | 41223332 | G>T | ITPKC | nonsynonymous |
| 19q13.31 | 44832258 | C>T | ZFP112 | synonymous |
| 19q13.41 | 52091670 | G>C | ZNF175 | nonsynonymous |
| 19q13.42 | 53740852 | T>C | ZNF677 | synonymous |
| 19q13.42 | 53856536 | A>G | ZNF845 | nonsynonymous |
| 20q11.22 | 33334691 | T>A | NCOA6 | nonsynonymous |
| 20q12 | 37621059 | G>T | DHX35 | nonsynonymous |
| 20q13.12 | 44048792 | G>T | PIGT | nonsynonymous |
| 20q13.12 | 44444531 | G>A | UBE2C | nonsynonymous |
| 20q13.32 | 57611612 | C>T | SLMO2 | nonsynonymous |
| 20q13.33 | 61959433 | C>G | COL20A1 | synonymous |
| 21q22.11 | 31691851 | C>A | KRTAP26-1 | nonsynonymous |
| 21q22.11 | 34635244 | G>A | IFNAR2 | synonymous |
| 21q22.2 | 40652109 | G>A | BRWD1 | synonymous |
| 22q12.1 | 29095914 | C>T | CHEK2 | nonsynonymous |
| 22q12.3 | 36537719 | T>C | APOL3 | synonymous |
| 22q13.1 | 38894124 | T>G | DDX17 | nonsynonymous |
| Xp22.2 | 16142325 | T>A | GRPR | synonymous |
| Xp21.1 | 34148240 | A>T | FAM47A | nonsynonymous |
| Xp11.22 | 51488285 | G>A | GSPT2 | synonymous |
| Xp11.21 | 54823489 | G>T | ITIH6 | nonsynonymous |
| Xp11.21 | 54823498 | G>T | ITIH6 | nonsynonymous |
| Xq13.1 | 69713276 | A>G | DLG3 | synonymous |
| Xq26.3 | 135430587 | T>A | GPR112 | synonymous |
| Xq28 | 148037741 | A>T | AFF2 | synonymous |

| **Non-Tumor-specific variants** | | | | |
| --- | --- | --- | --- | --- |
| 1p36.21 | 16199373 | C>G | SPEN | nonsynonymous |
| 1p36.13 | 16770147 | A>C | NECAP2 | nonsynonymous |
| 1p36.13 | 19528276 | G>C | UBR4 | stopgain |
| 1p34.2 | 43119647 | C>T | CCDC30 | nonsynonymous |
| 1p31.1 | 75684186 | T>C | SLC44A5 | synonymous |
| 1p22.1 | 93091413 | C>T | EVI5 | nonsynonymous |
| 1p21.2 | 99762305 | A>G | LPPR4 | synonymous |
| 1p21.2 | 100376388 | T>C | AGL | nonsynonymous |
| 1p13.3 | 108681808 | T>C | SLC25A24 | nonsynonymous |
| 1q21.3 | 153615726 | C>G | CHTOP | nonsynonymous |
| 1q22 | 156255110 | G>C | TMEM79 | synonymous |
| 1q22 | 156351667 | C>T | RHBG | nonsynonymous |
| 1q23.1 | 158450548 | T>G | OR10R2 | nonsynonymous |
| 1q24.2 | 169930306 | T>C | KIFAP3 | nonsynonymous |
| 1q24.3 | 171509923 | G>A | PRRC2C | synonymous |
| 1q25.3 | 182615921 | C>T | RGS8 | synonymous |
| 1q31.1 | 186106727 | T>C | HMCN1 | synonymous |
| 1q31.3 | 197070082 | C>G | ASPM | nonsynonymous |
| 1q32.3 | 213436143 | G>C | RPS6KC1 | nonsynonymous |
| 1q41 | 216538356 | A>G | USH2A | synonymous |
| 1q42.2 | 231401498 | G>A | GNPAT | nonsynonymous |
| 2p16.3 | 50463989 | C>T | NRXN1 | nonsynonymous |
| 2p11.2 | 84838960 | C>G | DNAH6 | nonsynonymous |
| 2q14.2 | 120725525 | C>G | PTPN4 | nonsynonymous |
| 2q21.3 | 135920325 | C>T | RAB3GAP1 | synonymous |
| 2q31.1 | 170782189 | C>A | UBR3 | nonsynonymous |
| 2q31.2 | 179593423 | T>C | TTN | synonymous |
| 2q32.1 | 183595807 | C>G | DNAJC10 | nonsynonymous |
| 2q32.1 | 187501807 | T>G | ITGAV | nonsynonymous |
| 2q33.3 | 207509344 | G>C | LOC200726 | synonymous |
| 2q35 | 217055046 | G>A | XRCC5 | nonsynonymous |
| 2q36.3 | 228476164 | C>T | C2orf83 | synonymous |
| 3p26.3 | 1189722 | G>C | CNTN6 | synonymous |
| 3p25.1 | 15253620 | C>A | CAPN7 | nonsynonymous |
| 3p25.1 | 15604885 | G>T | HACL1 | nonsynonymous |
| 3p22.2 | 37107365 | T>C | LRRFIP2 | synonymous |
| 3p21.31 | 44684731 | G>A | ZNF197 | synonymous |
| 3p21.2 | 51394463 | T>C | DOCK3 | nonsynonymous |
| 3p21.1 | 53139768 | A>G | RFT1 | nonsynonymous |
| 3p14.2 | 62142869 | C>T | PTPRG | nonsynonymous |
| 3q12.1 | 99509830 | G>A | COL8A1 | nonsynonymous |
| 3q13.2 | 111312537 | T>C | ZBED2 | nonsynonymous |
| 3q13.2 | 113379873 | G>T | KIAA2018 | nonsynonymous |
| 3q13.33 | 119209495 | G>A | POGLUT1 | nonsynonymous |
| 3q21.3 | 127774579 | C>T | SEC61A1 | synonymous |
| 3q21.3 | 129152940 | G>T | MBD4 | nonsynonymous |
| 4p15.31 | 17805221 | G>C | DCAF16 | nonsynonymous |
| 4p14 | 39891879 | C>T | PDS5A | nonsynonymous |
| 4p11 | 48512090 | C>T | FRYL | nonsynonymous |
| 4q21.22 | 83347225 | T>G | HNRPDL | nonsynonymous |
| 4q24 | 106197148 | G>C | TET2 | nonsynonymous |
| 4q24 | 106473935 | G>A | ARHGEF38 | nonsynonymous |
| 4q26 | 120085449 | C>A | MYOZ2 | nonsynonymous |
| 4q32.2 | 162307028 | C>A | FSTL5 | nonsynonymous |
| 4q35.2 | 187534404 | G>T | FAT1 | nonsynonymous |
| 5p13.3 | 32229952 | T>C | MTMR12 | nonsynonymous |
| 5p13.2 | 34028972 | G>A | C1QTNF3 | nonsynonymous |
| 5p13.2 | 34913678 | T>C | RAD1 | synonymous |
| 5p13.1 | 38502745 | C>T | LIFR | nonsynonymous |
| 5p13.1 | 38502747 | G>T | LIFR | nonsynonymous |
| 5p13.1 | 38954943 | T>G | RICTOR | nonsynonymous |
| 5p13.1 | 41018514 | C>A | HEATR7B2 | nonsynonymous |
| 5q11.2 | 54327293 | A>G | GZMK | synonymous |
| 5q13.2 | 73153514 | A>C | ARHGEF28 | synonymous |
| 5q14.1 | 77477409 | C>T | AP3B1 | synonymous |
| 5q14.1 | 79837622 | C>A | FAM151B | nonsynonymous |
| 5q14.1 | 80040411 | G>C | MSH3 | synonymous |
| 5q15 | 93120193 | C>T | FAM172A | nonsynonymous |
| 5q15 | 94936591 | C>A | ARSK | stopgain |
| 5q15 | 96503653 | G>T | RIOK2 | stopgain |
| 5q23.1 | 118456823 | A>C | DMXL1 | nonsynonymous |
| 5q23.2 | 121776345 | C>T | SNCAIP | nonsynonymous |
| 5q31.1 | 134191064 | T>G | C5orf24 | nonsynonymous |
| 5q33.2 | 153382488 | G>T | FAM114A2 | synonymous |
| 6p22.3 | 17764962 | G>T | KIF13A | stopgain |
| 6p22.1 | 29640438 | C>A | ZFP57 | nonsynonymous |
| 6p21.33 | 31498921 | G>T | DDX39B | nonsynonymous |
| 6p21.31 | 34846438 | C>T | TAF11 | nonsynonymous |
| 6p21.31 | 36294451 | G>T | C6orf222 | nonsynonymous |
| 6p12.3 | 49459959 | G>A | CENPQ | nonsynonymous |
| 6q13 | 73904577 | A>G | KCNQ5 | nonsynonymous |
| 6q22.1 | 117130644 | C>G | GPRC6A | nonsynonymous |
| 6q22.1 | 117842712 | C>T | DCBLD1 | nonsynonymous |
| 6q23.2 | 133032933 | T>C | VNN1 | nonsynonymous |
| 6q25.2 | 152589255 | C>A | SYNE1 | nonsynonymous |
| 6q25.2 | 153315773 | C>T | MTRF1L | nonsynonymous |
| 7p14.1 | 37924829 | G>A | NME8 | nonsynonymous |
| 7q11.23 | 72755276 | G>T | FKBP6 | nonsynonymous |
| 7q21.13 | 89793954 | T>C | STEAP1 | nonsynonymous |
| 7q21.13 | 89915636 | G>A | C7orf63 | nonsynonymous |
| 7q21.2 | 91624927 | G>A | AKAP9 | nonsynonymous |
| 7q21.2 | 91709105 | C>G | AKAP9 | nonsynonymous |
| 7q21.3 | 95775940 | T>C | SLC25A13 | synonymous |
| 7q36.1 | 148463663 | G>C | CUL1 | nonsynonymous |
| 8p21.2 | 24304723 | T>C | ADAM7 | nonsynonymous |
| 8p12 | 30925779 | T>G | WRN | synonymous |
| 8q13.1 | 67970390 | G>C | COPS5 | nonsynonymous |
| 8q21.13 | 82591439 | G>C | IMPA1 | nonsynonymous |
| 8q22.1 | 95523783 | C>G | KIAA1429 | nonsynonymous |
| 8q23.1 | 110489611 | A>C | PKHD1L1 | synonymous |
| 9p24.1 | 5044436 | C>G | JAK2 | nonsynonymous |
| 9p24.1 | 5922839 | T>G | KIAA2026 | nonsynonymous |
| 9p22.1 | 19063561 | G>A | HAUS6 | nonsynonymous |
| 9p21.3 | 20953042 | C>T | FOCAD | synonymous |
| 9p21.3 | 21077568 | T>C | IFNB1 | nonsynonymous |
| 9p13.3 | 33944421 | T>G | UBAP2 | nonsynonymous |
| 9p13.2 | 36583647 | G>C | MELK | nonsynonymous |
| 9q21.32 | 84235367 | A>G | TLE1 | nonsynonymous |
| 9q22.31 | 94809915 | A>C | SPTLC1 | nonsynonymous |
| 9q31.1 | 106877022 | C>T | SMC2 | nonsynonymous |
| 9q31.2 | 110087230 | G>A | RAD23B | nonsynonymous |
| 9q32 | 114989719 | A>G | PTBP3 | nonsynonymous |
| 9q32 | 116093304 | G>C | WDR31 | nonsynonymous |
| 9q33.2 | 125487088 | G>T | OR1L4 | nonsynonymous |
| 9q34.11 | 131247686 | A>G | ODF2 | nonsynonymous |
| 10p15.1 | 5789172 | C>A | FAM208B | nonsynonymous |
| 10p12.1 | 27700856 | T>C | PTCHD3 | synonymous |
| 10q21.2 | 61832638 | G>A | ANK3 | synonymous |
| 10q21.2 | 62544513 | G>T | CDK1 | nonsynonymous |
| 10q24.31 | 102286166 | T>G | NDUFB8 | nonsynonymous |
| 10q25.2 | 112360822 | A>C | SMC3 | nonsynonymous |
| 10q26.13 | 127422293 | G>T | C10orf137 | nonsynonymous |
| 11p15.4 | 4929199 | C>T | OR51A7 | synonymous |
| 11p15.4 | 5344968 | C>A | OR51B2 | nonsynonymous |
| 11p15.1 | 18637478 | T>G | SPTY2D1 | nonsynonymous |
| 11q12.1 | 55761278 | G>A | OR5F1 | nonsynonymous |
| 11q12.1 | 56020253 | C>A | OR5T3 | nonsynonymous |
| 11q12.1 | 56043638 | C>A | OR5T1 | nonsynonymous |
| 11q12.1 | 59575222 | T>G | MRPL16 | synonymous |
| 11q12.3 | 63064889 | T>G | SLC22A10 | synonymous |
| 11q13.1 | 65790473 | A>C | CATSPER1 | nonsynonymous |
| 11q13.4 | 73872592 | A>C | C2CD3 | nonsynonymous |
| 11q14.1 | 78277317 | T>C | NARS2 | nonsynonymous |
| 11q22.3 | 103339328 | G>T | DYNC2H1 | nonsynonymous |
| 12p13.31 | 9002303 | G>A | A2ML1 | synonymous |
| 12p12.1 | 24982791 | G>T | BCAT1 | nonsynonymous |
| 12q13.2 | 55641827 | C>G | OR6C74 | nonsynonymous |
| 12q21.31 | 81741484 | C>T | PPFIA2 | nonsynonymous |
| 12q23.1 | 98941545 | T>G | TMPO | nonsynonymous |
| 12q23.1 | 101381359 | G>A | ANO4 | synonymous |
| 12q24.13 | 112691893 | C>G | HECTD4 | nonsynonymous |
| 12q24.21 | 116460329 | T>G | MED13L | nonsynonymous |
| 12q24.31 | 124191321 | C>G | TCTN2 | nonsynonymous |
| 13q12.12 | 25374620 | T>C | RNF17 | nonsynonymous |
| 13q13.1 | 33250067 | C>A | PDS5B | nonsynonymous |
| 13q14.13 | 46276956 | G>C | SPERT | nonsynonymous |
| 13q14.3 | 51948537 | T>C | INTS6 | synonymous |
| 13q22.3 | 77673117 | T>G | MYCBP2 | nonsynonymous |
| 13q31.1 | 80125222 | T>G | NDFIP2 | synonymous |
| 13q33.1 | 102378975 | G>T | FGF14 | synonymous |
| 13q33.1 | 103389556 | G>T | CCDC168 | stopgain |
| 13q34 | 114156109 | C>G | TMCO3 | nonsynonymous |
| 14q12 | 31358852 | G>T | COCH | nonsynonymous |
| 14q13.2 | 36153095 | C>T | RALGAPA1 | nonsynonymous |
| 14q13.2 | 36154226 | A>G | RALGAPA1 | synonymous |
| 14q21.1 | 39591708 | A>C | GEMIN2 | synonymous |
| 14q23.1 | 59757965 | G>A | DAAM1 | nonsynonymous |
| 14q23.1 | 60712590 | G>C | PPM1A | nonsynonymous |
| 14q23.1 | 60712592 | A>G | PPM1A | synonymous |
| 14q23.3 | 65260331 | C>T | SPTB | nonsynonymous |
| 14q32.2 | 99929885 | T>C | SETD3 | nonsynonymous |
| 15q15.1 | 42158295 | G>A | SPTBN5 | nonsynonymous |
| 15q15.3 | 44177948 | T>C | FRMD5 | nonsynonymous |
| 15q24.3 | 77067363 | C>T | SCAPER | nonsynonymous |
| 15q25.1 | 79186431 | A>G | MORF4L1 | nonsynonymous |
| 16p13.3 | 2821524 | C>T | TCEB2 | synonymous |
| 16q12.1 | 47485331 | T>G | ITFG1 | nonsynonymous |
| 16q23.1 | 74335529 | G>A | PSMD7 | nonsynonymous |
| 16q24.3 | 89776279 | C>T | VPS9D1 | nonsynonymous |
| 17p13.1 | 6524317 | G>C | KIAA0753 | nonsynonymous |
| 17p13.1 | 10533169 | G>C | MYH3 | nonsynonymous |
| 17q11.2 | 29663798 | C>G | NF1 | nonsynonymous |
| 17q12 | 35548174 | G>T | ACACA | nonsynonymous |
| 17q21.31 | 41122327 | T>G | PTGES3L | synonymous |
| 17q23.2 | 59763503 | G>C | BRIP1 | nonsynonymous |
| 17q24.3 | 67197651 | C>T | ABCA10 | nonsynonymous |
| 17q24.3 | 67293342 | C>T | ABCA5 | nonsynonymous |
| 18p11.32 | 198072 | C>G | USP14 | nonsynonymous |
| 18q21.32 | 57136721 | C>T | CCBE1 | synonymous |
| 18q21.33 | 59739926 | G>C | PIGN | nonsynonymous |
| 18q21.33 | 61585292 | A>C | SERPINB10 | nonsynonymous |
| 18q22.1 | 65180183 | C>T | DSEL | nonsynonymous |
| 19p13.2 | 9021175 | G>C | MUC16 | nonsynonymous |
| 19p13.2 | 11541549 | C>T | CCDC151 | synonymous |
| 19p13.2 | 12256835 | C>T | ZNF625 | synonymous |
| 19q13.11 | 34710633 | G>A | LSM14A | synonymous |
| 19q13.43 | 56733492 | C>T | ZSCAN5A | nonsynonymous |
| 19q13.43 | 57840426 | G>A | ZNF543 | synonymous |
| 19q13.43 | 57868518 | C>T | ZNF304 | synonymous |
| 20p13 | 4680208 | T>G | PRNP | synonymous |
| 20p12.3 | 5157344 | C>A | CDS2 | stopgain |
| 20q12 | 37621059 | G>T | DHX35 | nonsynonymous |
| 20q12 | 40083292 | G>A | CHD6 | synonymous |
| 20q13.32 | 57611612 | C>T | SLMO2 | nonsynonymous |
| 22q13.2 | 42606460 | T>G | TCF20 | nonsynonymous |
| 22q13.33 | 50566890 | C>A | MOV10L1 | nonsynonymous |
| 22q13.33 | 50659571 | T>G | TUBGCP6 | nonsynonymous |
| Xp22.2 | 15793409 | C>A | CA5B | nonsynonymous |
| Xp11.22 | 50037896 | G>A | CCNB3 | nonsynonymous |
| Xp11.21 | 55513595 | C>A | USP51 | nonsynonymous |
| Xq21.1 | 84526138 | G>A | ZNF711 | synonymous |
| Xq22.3 | 104984624 | A>C | IL1RAPL2 | nonsynonymous |
