# Supplementary material for: Profiling of Exome Mutations Associated with Progression of HBV-Related Hepatocellular Carcinoma
Source: PLoS One. 2014 Dec 18;9(12):e115152. doi: 10.1371/journal.pone.0115152 (PMC4270755; doi:10.1371/journal.pone.0115152)
Supplement: S3 Table — Functional enrichment of non-tumoral specific variants. (DOCX) [file pone.0115152.s007.docx]

## Table S3. Functional enrichment of non-tumoral specific variants

|  | **Term** | **Gene**  **Count** | **P-Value** | **Genes** |
| --- | --- | --- | --- | --- |
| cell cycle | GO:0022403~cell cycle phase | 12 | 0.0045 | RAD1, BCAT1, CDK1, HAUS6, CCNB3, PDS5B, PDS5A, TTN, SMC2, SMC3, ASPM, CUL1 |
|  | GO:0000278~mitotic cell cycle | 11 | 0.0059 | BCAT1, CDK1, HAUS6, PDS5B, PDS5A, TTN, PSMD7, SMC2, SMC3, ASPM, CUL1 |
|  | GO:0000279~M phase | 10 | 0.0083 | RAD1, CDK1, HAUS6, CCNB3, PDS5B, PDS5A, TTN, SMC2, SMC3, ASPM |
|  | GO:0007067~mitosis | 8 | 0.0089 | CDK1, HAUS6, PDS5B, PDS5A, TTN, SMC2, SMC3, ASPM |
|  | GO:0051301~cell division | 9 | 0.0131 | CDK1, HAUS6, CCNB3, PDS5B, PDS5A, EVI5, SMC2, SMC3, ASPM |
|  | GO:0022402~cell cycle process | 13 | 0.0166 | BCAT1, CDK1, HAUS6, PDS5B, PDS5A, TTN, SMC2, SMC3, RAD1, CCNB3, PSMD7, CUL1, ASPM |
|  | GO:0007049~cell cycle | 15 | 0.0351 | BCAT1, CDK1, HAUS6, PDS5B, PDS5A, TET2, TTN, SMC2, SMC3, RAD1, CCNB3, EVI5, PSMD7, CUL1, ASPM |
| DNA repair | GO:0006281~DNA repair | 9 | 0.0106 | RAD1, XRCC5, MORF4L1, RAD23B, MSH3, BRIP1, MBD4, WRN, SMC3 |
|  | GO:0006974~response to DNA damage stimulus | 10 | 0.0176 | RAD1, XRCC5, MORF4L1, RAD23B, CDK1, MSH3, BRIP1, MBD4, WRN, SMC3 |
| morphogenesis | GO:0048858~cell projection morphogenesis | 8 | 0.0153 | C2CD3, LPPR4, ANK3, DYNC2H1, LIFR, JAK2, NRXN1, MYCBP2 |
